# Sex and Region-Specific Differences in Microglial Morphology and Function Across Development

**DOI:** 10.3390/neuroglia6010002

**Published:** 2025-01-04

**Authors:** Indra R. Bishnoi, Evan A. Bordt

**Affiliations:** Department of Pediatrics, Lurie Center for Autism, Massachusetts General Hospital, Harvard Medical School, Boston, MA 02129, USA

**Keywords:** age, baseline, brain regions, critical periods, mouse, rat, sex

## Abstract

Microglia are exceptionally dynamic resident innate immune cells within the central nervous system, existing on a continuum of morphologies and functions throughout their lifespan. They play vital roles in response to injuries and infections, clearing cellular debris, and maintaining neural homeostasis throughout development. Emerging research suggests that microglia are strongly influenced by biological factors, including sex, developmental stage, and their local environment. This review synthesizes findings on sex differences in microglial morphology and function in key brain regions, including the frontal cortex, hippocampus, amygdala, hypothalamus, basal ganglia, and cerebellum, across the lifespan. Where available, we examine how gonadal hormones influence these microglial characteristics. Additionally, we highlight the limitations of relying solely on morphology to infer function and underscore the need for comprehensive, multimodal approaches to guide future research. Ultimately, this review aims to advance the dialogue on these spatiotemporally heterogeneous cells and their implications for sex differences in brain function and vulnerability to neurological and psychiatric disorders.

## 1. Introduction

Microglia, the resident innate immune cells of the central nervous system (CNS), were independently discovered by Dr. Franz Nissl and Dr. William Ford Robertson and later studied in detail by Dr. Pío del Río Hortega [1]. Depending on the brain region, microglia make up about 5–12% of the adult brain and are the only cells in the brain that are not ‘born’ there [2]. These cells originate from embryonic yolk sac erythromyeloid progenitors, which migrate into the developing CNS before the closure of the blood–brain barrier, which begins around embryonic day (E) 9.5 in rodents [3,4]. While the timing of microglial colonization in humans is not as well-defined, it is predicted to occur between gestational weeks 4 and 24 [5,6].

In the CNS, microglia play vital roles in responding to injury and infection, clearing cellular debris, and maintaining neural homeostasis [7,8]. Beyond their classical immune functions, microglia are increasingly recognized for their involvement in brain development, neuro- and synaptogenesis, synaptic pruning, and neuroplasticity [7,9]. Emerging research suggests that the morphology and function of microglia are exceptionally dynamic and influenced by a variety of factors, including developmental stage, brain region, and sex.

Sex differences in microglial activity have garnered significant attention in recent years, driven by growing evidence that microglia in males and females exhibit distinct morphological and functional trajectories [10,11]. These differences may contribute to the sex-biased prevalence of several neurodevelopmental and neurodegenerative disorders, including autism spectrum disorder and Alzheimer’s disease [7,9,12]. Despite the implications of this work, the understanding of how microglial sex differences manifest across different brain regions and developmental stages in males and females remains limited.

This review summarizes our current understanding of sex differences in microglial characteristics, focusing on key brain regions, including the frontal cortex, hippocampus, amygdala, hypothalamus, basal ganglia, and cerebellum, across the lifespan of rodents. By integrating morphological and functional data, we highlight the complexities of the existing literature and emphasize the need for further investigations in this area of research. Ultimately, this review aims to advance the dialogue on sex differences in microglia and their broader implications for brain function and understanding sex-biased vulnerabilities for neurological and psychiatric disorders.

### 1.1. Gonadal Hormones Across Development

Gonadal hormones, like estrogens and androgens, aid in the establishment of sex-dependent effects across brain development, including guiding gene expression, cell development, migration, differentiation, apoptosis, and neural circuit formation [13,14]. While gonadal hormones aid in the organization of the brain and ‘activation’ of sex differences during critical periods in early life and adolescence [15,16], they can also alter physiology and behavior in adulthood [17].

**Figure 1.**
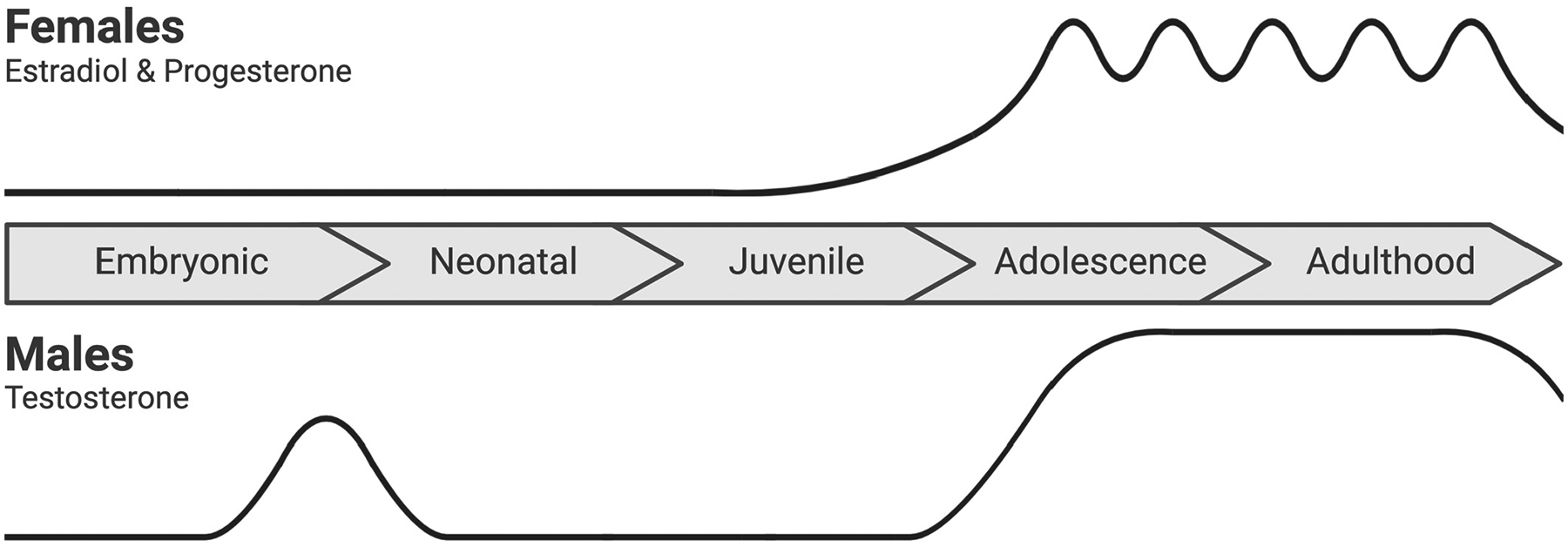
Gonadal hormone secretion across the embryonic (embryonic day 0 to birth), neonatal (postnatal day 0 to 10), juvenile (postnatal day 11 to 20), adolescent (postnatal day 21 to 59), and adulthood (postnatal day 60 onwards) developmental stages in mice and rats. In the late embryonic and early neonatal period, testosterone and its aromatized metabolite estradiol drive the defeminization and masculinization of males. In females, the relative absence of early gonadal hormone secretion allows for feminization. A second surge of gonadal hormones occurs during adolescence. Around P40, testosterone increases in males, and estradiol and progesterone increase in females, oscillating with the estrous cycle before declining in late adulthood. Created in BioRender. Bishnoi, I. (2024) https://BioRender.com/w25p782 (accessed on 3 December 2024).

At the end of the embryonic period and just after birth (perinatally), male testes produce testosterone [18], which is aromatized into estradiol across brain regions [19] (Figure 1). This exposure to early gonadal hormones drives the defeminization and masculinization of male rodents [20,21]. Our understanding of the mechanisms underlying the defeminization and masculinization of male rodents is growing, with some studies suggesting a mediating role of estrogen receptor alpha (ERα) and/or beta (ERβ) [20,21].

The next wave of gonadal hormones occurs around postnatal day (P) 40. At this stage, we see a surge of testosterone in males and estradiol and progesterone in female rodents [22,23]. After this surge, testosterone levels can dip slightly, after which they stabilize in males, and the estrous cycle begins in females [24] (Figure 1). During the estrous cycle, estradiol levels are highest during the proestrus phase, while progesterone peaks during the diestrus phase [25,26].

Microglia possess receptors for the major classes of steroid hormones, including ERα and ERβ, as well as G protein-coupled estrogen receptor 1 (GPER1), progesterone receptor (PR), and androgen receptor (AR) [27–29]. Further, microglia are increasingly found to play necessary roles in shaping the sex-specific trajectory of brain structure and function, as well as behavior [30–33]. Given this synergistic relationship between microglia and gonadal hormones, it is unsurprising that there are sex differences in microglial morphology and function. In this review, we discuss brain-region-specific sex differences in microglial morphology and function in mice and rats. Where possible, we highlight studies that investigate the roles of gonadal hormones in these sex differences and their behavioral outcomes.

### 1.2. Microglial Morphology and Function

Early studies of microglial reactivity often examined microglial density and the number of microglia in a specified area or categorized microglial morphology into two basic morphological phenotypes: ramified or amoeboid. These phenotypes were then coupled with their proposed functions. Ramified microglia were characterized as ‘resting’ or homeostatic/surveillant, while amoeboid microglia were thought to be ‘activated’ or phagocytic [34,35]. Ramified resting microglia were initially thought to remain quiescent until activated by injury and/or infection, at which point they would become amoeboid in shape and take on typical macrophage-like immune functions [34]. Once activated, microglia were categorized as proinflammatory (M1) or anti-inflammatory (M2). This terminology was adopted from the macrophage research literature, which regarded M1 microglia as neurotoxic and M2 microglia as neuroprotective [34]. While these strict classifications initially aided in our understanding of microglia, microglial functions are increasingly recognized to be far more heterogeneous. For example, we now understand that microglia in the healthy brain are not dormant. Instead, they are highly active, constantly sampling their microenvironment [36] and playing important roles in sculpting neural development [37–39].

As our understanding of microglial functions has evolved, it has become clear that many of our discussions regarding microglial functions are grounded in inferences made from microglial morphology. Yet, microglia exist along a continuum of morphologies. These morphologies can certainly change based on function. For example, ramified microglia in the developing brain express surveillant markers, but they can also express phagocytic markers [40,41]. These findings suggest that microglia may transition between multiple states throughout their lifespan [34,42,43].

What guides these transitions? Recent technological advancements have revealed that microglial morphology is influenced by factors such as sex and age [44]. Moreover, De Biase et al. [45] showed that even if microglia are experimentally ablated, the distinct microglial morphology established in the basal ganglia during early development can re-emerge in adulthood, guided by cues from the local environment. This underscores the importance of the microenvironment in shaping microglial morphology across development [45,46]. Additional factors, such as circadian rhythms, also affect microglial morphology [47,48]. Thus, although microglial morphology provides insights into their function, it does not necessarily equate to microglial function. Consequently, relying solely on morphology as an indicator of microglial function may be insufficient. This review presents and compares morphological and functional evidence for microglial sex differences where available and highlights areas in which further research is needed.

## 2. Brain Region-Specific Microglial Morphology and Function

In this review, we provide an overview of the region- and sex-specific morphological and functional characteristics of microglia that fluctuate across development. Given the variation in the methodologies and analyses used in the discussed papers, it is presently challenging to draw overarching conclusions between brain regions. Instead, here we independently synthesize research on microglia within the frontal cortex, hippocampus, amygdala, hypothalamus, basal ganglia, and cerebellum.

### 2.1. The Frontal Cortex

The frontal cortex is a multifunctional brain region engaged in cognitive control, working memory, motor control, social interaction, and emotional processing [49]. Microglia within the frontal cortex are increasingly recognized for their roles in cognition and emotional regulation [50–53]. However, our understanding of the baseline morphologies and functions of microglia within this region and how these properties change during development and across sexes remains limited.

In early development (P6 to P15), microglial density increases dramatically in the anterior cingulate cortex (ACC) of both sexes of C57BL/6J mice in sex-matched groups [54]. Additionally, the proportion of phagocytic microglia decreases by P15 in the ACC of these mice [54]. By adolescence (P20-P21 and P40), studies in Sprague Dawley rats and C57BL/6J mice indicate no sex differences in microglial density in the frontal or prefrontal cortex (PFC) [55,56]. Nevertheless, Gildawie et al. [56] observed sex differences in microglial morphology in Sprague Dawley rats within the PFC. The microglia of females exhibited larger soma sizes than males at P20, while the microglia of males had larger soma sizes than females at P40 [56]. Soma size is a morphological marker that has been used to infer activation status. An enlargement of the soma is traditionally associated with heightened microglial responsiveness to stimuli. This morphological change can be accompanied by reduced branching as microglia take on a more amoeboid shape [57,58]. Gildawie et al. [56] observed that microglia in female rats exhibited greater microglial branching compared to males at both P20 and P40. These findings suggest that the microglia of females may be transitioning to a more ramified or surveillance-oriented state from the juvenile to the adolescent period of development [57]. Whether these sex-specific morphological shifts correspond to functional disparities, such as variations in phagocytic activity, requires further study.

Adolescence is a time of significant changes in the frontal cortex. During this critical period, the PFC undergoes maturation, which is essential for developing functions like higher-order cognition [59,60]. Microglia have been found to phagocytose both pre- and post-synaptic elements of neurons in adolescence [61] without a significant change in microglial density and morphology during this process [62]. Synaptic refinement by microglia is critical in adolescence. Indeed, experimentally depleting microglia at this age perturbs synaptic pruning and disrupts cognition in both male and female adult C57BL6/N mice [63,64]. Some of these disruptions in cognition are sexually dimorphic, such as an increased expression of contextual fear conditioning in females but not males [63,64].

Adolescence also marks the stage at which estradiol levels rise in female rodents and testosterone surges in males [22,23]. Estradiol is thought to exert neuroprotective or anti-inflammatory effects on microglia, dampening their immune response by reducing nitric oxide production, proinflammatory genes, proinflammatory cytokine release, and phagocytic activity [65]. These effects may promote a stunted immune response, lower neurotoxicity upon immune activation, and/or lead to a bias toward maintaining homeostasis in female microglia. Indeed, in addition to the ramified morphology of microglia in adolescent females [56], the microglia of adult (P75) female Sprague Dawley rats are more ramified in the PFC than males [66]. Importantly, the latter findings were coupled with greater CX3CL1–CX3CR1 expression in females compared to males [66]. Signaling between fractalkine (CX3CL1) from neurons and its receptor (CX3CR1) on microglia mediates several microglial functions, though it is typically known for its anti-inflammatory or neuroprotective role in the CNS [67–69]. Furthermore, C57BL/6J female mice exhibit a smaller soma size compared to males at P91 [55], a morphological measure that potentially indicates lower phagocytic activity. Finally, Barko et al. [70] found 982 differentially expressed transcripts between microglia isolated from the PFC of male and female adult mice (534 of which were expressed at higher levels in males, and 448 were expressed at higher levels in females). Pathway analyses of these transcripts revealed that female PFC microglia may be more involved in cellular maintenance and repair than male prefrontal microglia, which may prioritize synaptic regulation [70]. Together, these studies suggest that frontal microglia in adolescent and adult female rodents exhibit a ramified morphology and may potentially engage in anti-inflammatory or homeostatic functions. Whether this sex difference is mediated by estradiol remains to be directly tested.

In contrast, microglia in the frontal cortex of males display heightened immune reactivity during adulthood. At P91, microglia in males display greater antigen presentation capacity than females, as evidenced by an increased expression of major histocompatibility complex (MHC) class I and II molecules [55]. This increased antigen presentation capacity suggests that microglia in males may be primed to respond to stimuli [71,72]. Moreover, electrophysiological responses of microglia in C57BL/6J male mice have higher baseline currents and a stronger response to ATP, which can act as a signaling molecule for microglia during immunological challenge [55]. Proteomic analyses further support this enhanced reactivity in males, revealing a higher expression of P2X receptors P2X4 and P2X7 in male C57BL/6J mice compared to females [55]. Both P2X4 and P2X7 receptors help microglia detect extracellular ATP as a danger signal to trigger an immune response [73]. As such, these findings suggest that microglia in the frontal cortex of males may be more likely to mount a heightened response to challenges, like infections, compared to females.

**Figure 2.**
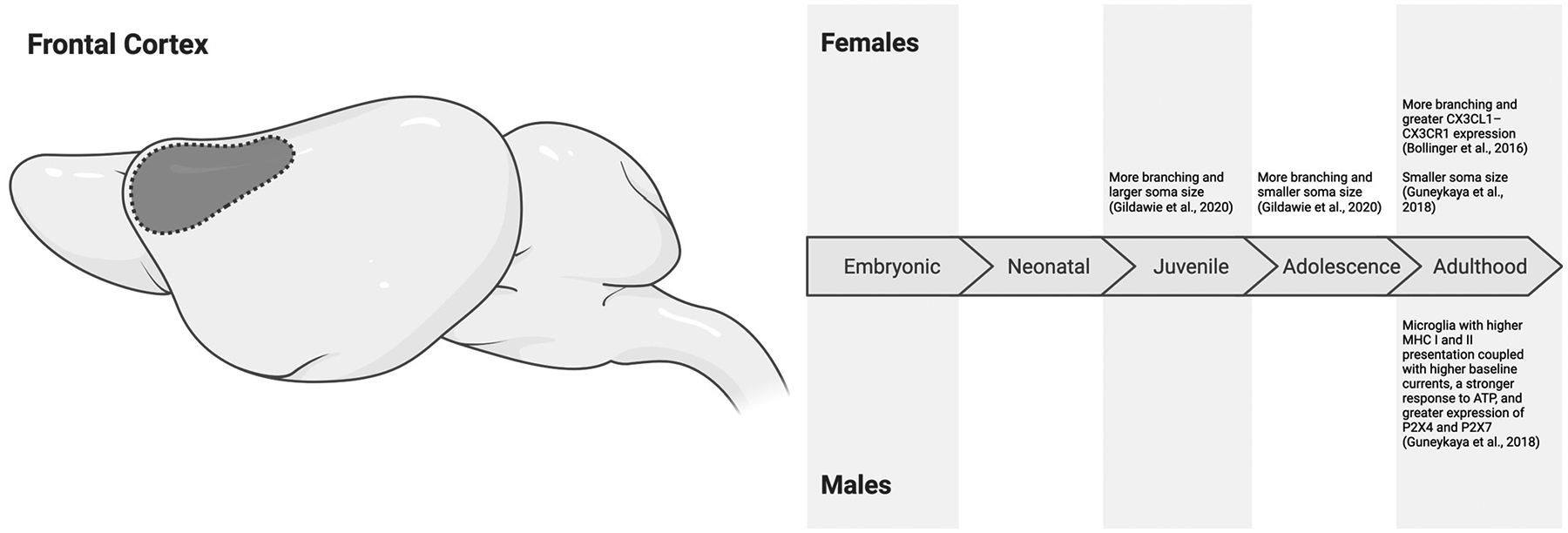
Sex differences in microglial characteristics across the embryonic (embryonic day 0 to birth), neonatal (postnatal day 0 to 10), juvenile (postnatal day 11 to 20), adolescent (postnatal day 21 to 59), and adulthood (postnatal day 60 onwards) developmental stages in the frontal cortex of mice and rats. All effects are in comparison to opposite-sex conspecifics. While we are starting to uncover sex differences in microglial morphology during the juvenile and adolescent periods [56], during adulthood, females may exhibit more ramified microglia that may prioritize anti-inflammatory or homeostatic functions [55,66]. In contrast, microglial characteristics in adult male rodents may show a bias for heightened immune reactivity in the frontal cortex [55,66]. Created in BioRender. Bishnoi, I. (2024) https://BioRender.com/r88b180 (accessed on 3 December 2024).

In summary, there is limited research on embryonic and neonatal microglial morphology and function in the frontal cortex. Additionally, studies do not presently support consistent sex differences during the juvenile period. During adolescence, frontal microglia engage in synaptic refinement, thereby altering adulthood behaviors. In adolescence and adulthood, females tend to exhibit more ramified microglia that may prioritize surveillance-oriented or homeostatic functions compared to male conspecifics. Conversely, the microglia of males demonstrate a bias towards heightened immune reactivity during adolescence and adulthood (Figure 2). These differences appear to align with the gonadal hormonal surges of adolescence, though the mechanisms driving these changes remain unclear. Future studies exploring the roles of frontal microglia should focus on the interplay between hormones and microglial function to better understand the emergence of these sex-specific patterns and their effects on behavioral endpoints.

### 2.2. The Hippocampus

The hippocampus is integral to spatial navigation and the encoding of episodic memories, particularly recollection [74]. Microglia within the hippocampus have been extensively studied for their contributions to adult neurogenesis [75], as well as their roles in neurodegenerative and psychiatric disorders such as Alzheimer’s disease and depression [76,77]. More research has been conducted on sex differences in microglial morphology and function in the hippocampus compared to any other brain region. Despite these advancements, significant gaps remain in our knowledge.

Research on microglial morphology in the hippocampus has uncovered intriguing developmental patterns that vary by sex and subregion. Schwarz et al. [78] explored these variations across the cornu ammonis 1 (CA1), CA3, and the dentate gyrus (DG) in Sprague Dawley rats. While no sex differences in microglial morphology were observed at E17, amoeboid microglia and those with short branches (or stout processes) predominated over ramified microglia (microglia with longer or thinner branches/processes) at this stage. Sex differences began to emerge by birth (P0), with females exhibiting a greater density of microglia, particularly amoeboid microglia, and those with short branches than males in the CA1 and CA3 regions. Additionally, males had a significantly higher density of microglia in the CA3 region than females. Thus, although Schwarz et al. [78] found no prenatal sex differences at E17, they observed dynamic, hippocampal subregion-specific sex differences in microglial morphology in the neonatal period from birth through P4.

Other studies of neonatal microglia have reported contrasting findings in the same rat species. Nelson et al. [79,80] found no sex differences in microglial density in the hippocampus. However, they observed a higher number of phagocytic cups in the DG and the molecular layer of the hippocampus in females compared to males at P2/P3. The presence of phagocytic cups—structures formed during the engulfment of apoptotic cells and cellular debris—can serve as an indicator of microglial activity [80,81]. Thus, these findings suggest that microglia in neonatal females may exhibit greater phagocytic activity than males. These findings were corroborated by higher expression levels of phagocytic genes such as CD68, Cybb, Trem2, and Tyrobp in females, aligning well with the observed differences in phagocytic cups. Indeed, Trem2-deficient mice are unable to form phagocytic cups [82]. Nelson et al. [79] also explored other functional markers like C1q, C3, Mfge8, Gpr34, Itgam (alias of CD11b/CD18), Cx3cr1, and P2ry12, but did not find significant sex differences in their expression. These markers are commonly associated with various microglial functions, including complement pathways (e.g., C1q, C3) and microglial homeostasis (e.g., Cx3cr1, P2ry12). The lack of sex differences in these markers suggests that while phagocytic activity may differ between sexes during the neonatal period, other microglial functions may remain relatively similar between sexes.

Estradiol appears to modulate microglial activity in the neonatal hippocampus. Nelson et al. [79] also demonstrated that treatment with exogenous estradiol to masculinize female rats significantly decreased the number of phagocytic microglia in the DG of the hippocampus on P3. Following estradiol treatment, the number of phagocytic microglia observed in masculinized females was comparable to that of males but significantly lower than the density observed in females. These effects were specific to treatment with estradiol, as treatment with dihydrotestosterone did not alter the number of phagocytic microglia (i.e., those with phagocytic cups) [79]. This finding suggests a potentially modulating role for estrogens in shaping microglial outcomes within the DG of the hippocampus during early postnatal development. Interestingly, hippocampal estrogen receptor expression peaks between P4 and P10 in both sexes [83,84], allowing for the possibility that these receptors may mediate estradiol’s effects on microglial function. However, whether these receptors or other downstream targets play a defining role in hippocampal sex differences in microglial characteristics is presently unclear.

By adolescence (P30), microglial morphology becomes more ramified, with fewer observable sex differences, at least in the CA1 region of the hippocampus [78]. In the CA3 and DG regions of the hippocampus, Schwarz et al. [78] demonstrated that microglia in females have thicker and longer branches than microglia in males, a pattern that persisted into adulthood (P60) across the studied hippocampal subregions (CA1, CA3, and DG) [78]. In adulthood, transcriptomic analyses have uncovered 324 genes expressed at higher levels and 867 genes at lower levels in males compared to female microglia in the hippocampus of C57BL/6J mice [55]. Subsequent pathway analysis suggested that the genes more highly expressed in female microglia are involved in pathways related to cellular integrity and homeostasis [55].

In addition to varying with age, sex differences in microglial characteristics such as density and morphology appear to vary with methodology. A study in C57BL/6NNia mice demonstrated a 25–40% greater density of microglia in the DG and CA1 of 3–4-month (P60–90), 13–14-month (P395–425), and 20–24-month (P608–730) female mice compared to males [85]. This sex difference grew larger with age [85]. However, Guneykaya et al. [55] reported a reduced total microglial density in 3-week-old (P21) and 13-week-old (P91) female C57BL/6J mice compared to males. This discrepancy may reflect differences in microglial labeling techniques. Mouton et al. [85] employed Macrophage-1+ (Mac-1+) labeling, while Guneykaya et al. [55] labeled Iba+ cells. While Iba staining typically requires using additional labels for greater specificity (e.g., Tmem119, CD45/CD11b, CD68, etc.) [86], it is more specific to microglia [87]. Mac-1 is a part of the complement receptor 3 consisting of CD11b and CD18. These receptors are expressed on a variety of myeloid cells, like microglia, but also peripherally derived myeloid cells, such as centrally infiltrating monocytes [88]. As infiltration increases during both pathology and with age [89], it is possible that Mouton et al. [85] observed an increase in myeloid cells, rather than microglia alone, in females across age.

Though Iba1 staining alone only identifies microglia, combining it with CD68 staining allows for the characterization of activated phagocytic microglia [51,90]. Using Iba1 and CD68 staining, Weinhard et al. [91] observed a greater number of phagocytic microglia in the CA1 region of the hippocampus in female C57BL/6J mice compared to males on P8. This sex difference disappeared by P15. Intriguingly, by P28, the pattern reversed, with males exhibiting a greater number of phagocytic microglia before the sex difference disappeared again at P40 [91]. By adulthood (P91), male mice exhibit a larger microglial soma size and higher MHC I presentation, suggesting that microglia in males may be primed to respond to stimuli (although MHC II presentation did not differ between sexes). Despite these differences, no sex differences were found in the resting membrane potentials of microglia in the hippocampus of C57BL/6J mice, suggesting similar baseline excitability [55].

**Figure 3.**
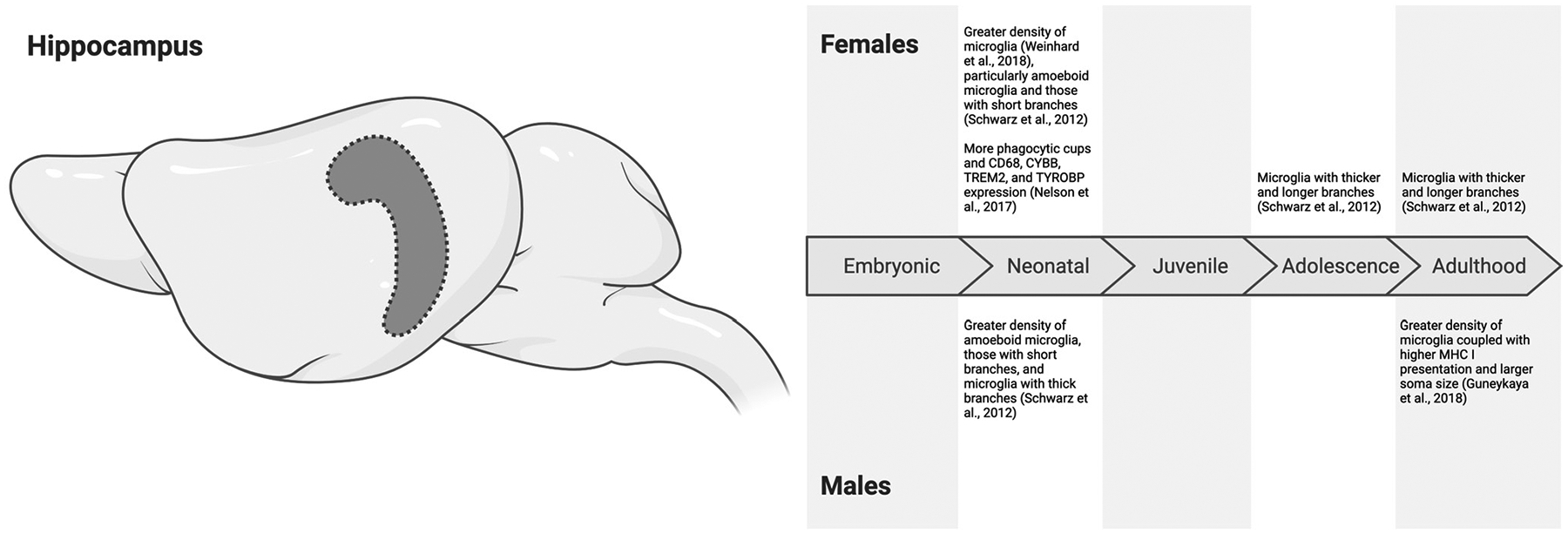
Sex differences in microglial characteristics across the embryonic (embryonic day 0 to birth), neonatal (postnatal day 0 to 10), juvenile (postnatal day 11 to 20), adolescent (postnatal day 21 to 59), and adulthood (postnatal day 60 onwards) developmental stages in the hippocampus of mice and rats. All effects are in comparison to opposite-sex conspecifics. During the neonatal period, the microglia of female rodents may be more phagocytic [78,79]. Microglia of females may transition into a more ramified state from adolescence to adulthood, while microglia of male rodents may take on a more phagocytic role within the hippocampus [55,78]. Created in BioRender. Bishnoi, I. (2024) https://BioRender.com/m04r955 (accessed on 3 December 2024).

Similar to the frontal cortex, there is a paucity of research conducted within the embryonic stage within the hippocampus. During the neonatal period, microglia of male rodents may display a decreased propensity for immune reactivity. This sex difference in the neonatal period may be driven by the masculinization we see at this stage in males [79]. A finding that is presumably in opposition to this—if microglial morphology and function were taken to be in alignment—is that we find more amoeboid-shaped microglia in males on P4 [78]. This would suggest that microglia in males should instead be more phagocytic [53]. As noted, microglial morphology is not synonymous with function and is often a lagging indicator. Indeed, while Nelson et al. [79] found significant sex differences in the functions of microglia when morphological differences across hippocampal regions were explored, they observed no significant differences between females, masculinized females, and males on P2/P3. This mismatch between microglial morphology and function may be especially relevant during early development and warrants further investigation. From adolescence into adulthood, microglia in females appear to transition into a more ramified state, while microglia in males may become more phagocytic (Figure 3). Importantly, the observed sex differences are often specific to subregions of the hippocampus. These findings emphasize the variability that may be observed even within a single brain region and reveal a need to refine our techniques to better capture this variability.

### 2.3. The Amygdala

The amygdala is traditionally associated with fear and anxiety regulation, but its roles extend to learning, memory, and social cognition, including play behaviors [92,93]. Recent findings have highlighted the involvement of microglia in several functions of the amygdala, particularly in shaping social play behaviors [39,92]. While research into sex differences in amygdalar microglia is still emerging, early evidence suggests these cells may contribute to masculinized and feminized behaviors.

During development, microglia migrate into the CNS and settle in the developing brain around E17. At this age, no significant sex differences in microglial morphology have been observed in the amygdala of male and female Sprague Dawley rats, with most microglia exhibiting amoeboid morphologies [78]. By birth (P0), interactions between sex and microglial morphology begin to emerge. In Sprague Dawley rats, Schwarz et al. [78] reported a greater number of amoeboid microglia with short branches in females, while VanRyzin et al. [39] found more phagocytic microglia in males, as indicated by the presence of phagocytic cups. This discrepancy highlights another mismatch between microglial morphological and more functional indicators, especially at earlier developmental timepoints. By P4, we see a greater alignment between morphology and more functional indicators in Sprague Dawley rats with greater amoeboid microglia and those with more phagocytic cups present in males compared to females [39,78]. These sex differences appear to be transient, dissipating by P6 [39,80] when microglial density in the CNS increases dramatically [94] (though it is unclear if this is true across brain regions).

Further investigations by VanRyzin et al. [39] explored microglial characteristics in vitro and revealed that sex differences in microglial activity in the amygdala depend on local cues, including gonadal hormones. As noted, testosterone is aromatized into estradiol across brain regions during the perinatal period [19] and has been implicated in the defeminization and masculinization of male rodents [20,21]. Thus, VanRyzin et al. [39] masculinized females with either a surge of exogenous testosterone or estradiol around P0. Using this paradigm, they demonstrated that testosterone, but not estradiol, increased the number of phagocytic microglia in masculinized females to levels observed in males on P4. The researchers were also able to demonstrate that these microglia were necessary for masculinizing social play behaviors in early adolescent (P26/P27) Sprague Dawley rats [39]. Together, this comprehensive study suggests that amygdalar microglia in males exhibit androgen-promoted increases in baseline phagocytic activity shortly after birth, which may guide the masculinization of behaviors driven by the amygdala, like social play.

In adolescence and adulthood (P30, P60), microglia in the amygdala continue to show intriguing morphological sex differences. Female Sprague Dawley rats have been observed with more microglia exhibiting long, thick branches than males by P30 and P60 [78]. In mice (C57BL/6J), females exhibit higher microglial density in the amygdala in early adolescence (P21) compared to male conspecifics, but this sex difference flips in adulthood at P91 [55]. While no sex-dependent effects are found during P21 in soma size, at P91, microglial soma size is much larger in males [55].

**Figure 4.**
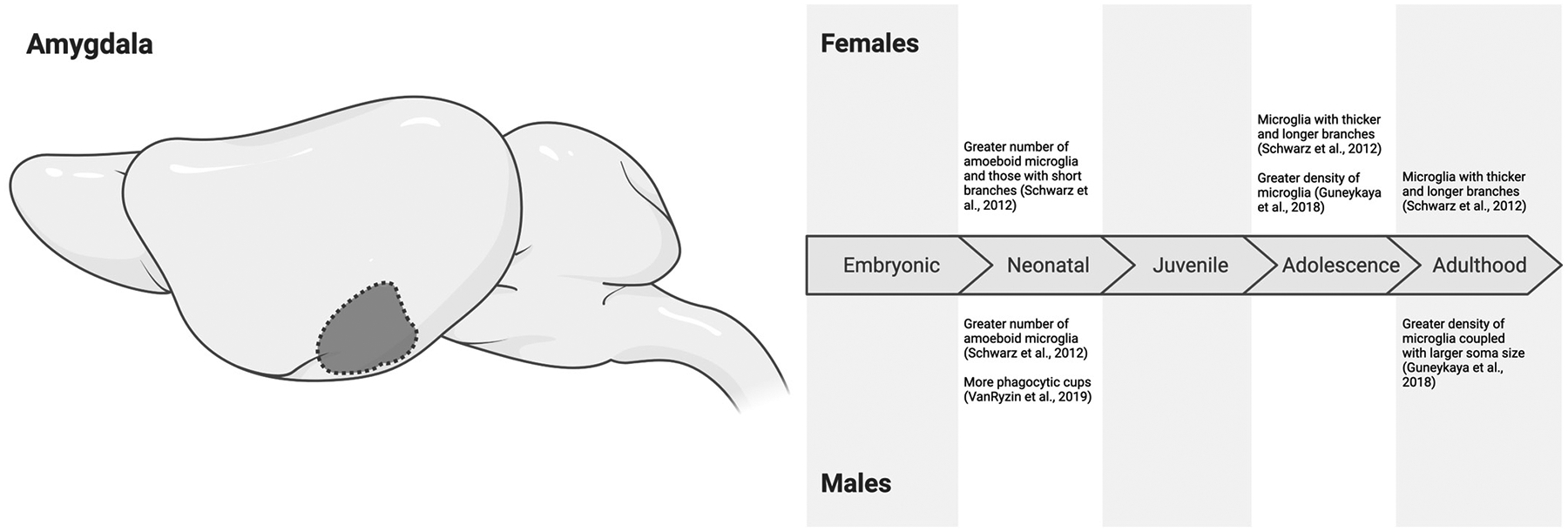
Sex differences in microglial characteristics across the embryonic (embryonic day 0 to birth), neonatal (postnatal day 0 to 10), juvenile (postnatal day 11 to 20), adolescent (postnatal day 21 to 59), and adulthood (postnatal day 60 onwards) developmental stages in the amygdala of mice and rats. All effects are in comparison to opposite-sex conspecifics. While the morphology and function of microglia during the neonatal period are more inconsistent, amygdalar microglia in females may prioritize an earlier transition toward a ramified state [39,78], which continues into adolescence and adulthood, while microglia in the male amygdala may retain more phagocytic roles [55,78]. Created in BioRender. Bishnoi, I. (2024) https://BioRender.com/o82o967 (accessed on 3 December 2024).

Thus, there is a growing consensus that amygdalar microglia in males exhibit more phagocytic activity in the neonatal period (P0–P4). Furthermore, we are learning that this sex-dependent difference may be guided by early androgens. By P6, these sex differences may subside, indicating that males have an earlier but transient increase in microglial activation (Figure 4). Based on morphology, the microglia of females may prioritize an earlier transition toward a homeostatic state, which continues into adolescence and adulthood, while microglia in the male amygdala may retain a heightened readiness for reactivity for a longer period. Functional assays are necessary to confirm whether or not these morphological indicators truly speak to sex differences in microglial function within the amygdala.

### 2.4. The Hypothalamus

The hypothalamus regulates endocrine and autonomic functions. It acts as a key coordinator between several brain regions to moderate the pituitary gland. Through this crosstalk, the hypothalamus maintains homeostasis, controls body temperature, regulates appetite, influences emotionality (such as social behaviors), and much more [95,96]. Within the hypothalamus, microglia have been implicated in diseases such as obesity [97]. Notably, the hypothalamus exhibits high aromatase activity across mammals [98,99]. As noted, this facilitates the conversion of testosterone to estradiol, a critical process in the masculinization and defeminization of hypothalamic structures such as the paraventricular nucleus (PVN) and preoptic area (POA).

During the embryonic period (E15.5), microglia of male and female CD1 mice present similar transcriptomic, protein, and protein secretion signatures within the hypothalamus [100]. In accordance with these findings, Schwarz et al. [78] observed no significant sex differences in microglial morphology in the PVN of the hypothalamus in Sprague Dawley rats at E17. However, Schwarz et al. [78] found more amoeboid microglia, as well as microglia with short branches, compared to those with long, thick, or thin branches in both sexes at this age. These findings do not necessarily imply that there are no sex differences in microglial characteristics during the embryonic period. Rather, sex differences may be latent, only emerging later in life or in the presence of a stimulus (e.g., a prenatal stressor) [100]. By birth (P0), Schwarz et al. [78] noted that male rats had a decline in amoeboid microglia and those with short branches, but these changes may have been transient as no significant sex-dependent morphological differences were detected on P4. In adolescence and adulthood (P30 and P60), microglia in the PVN began transitioning to a more ramified morphology, though again without clear sex differences [78].

In contrast to the PVN, the POA shows clearer evidence of sex differences in microglial morphology and function. At P2, male Sprague Dawley rats have more microglia in the POA, largely driven by an increase in amoeboid microglia with larger soma sizes and less branching compared to microglia in females [40]. The number of ramified and transitioning microglia did not significantly differ between sexes. Importantly, early masculinization and defeminization with estradiol treatment increased total and amoeboid microglia in females, in addition to increasing soma size and decreasing branching to levels seen in male conspecifics [40]. These findings highlight the role of estradiol-mediated masculinization and defeminization in microglial sex differences within the POA. Another notable finding by Lenz et al. [40] was that microglial cells at P2 were positive for IL-1β and TNFα, as well as CD206 co-labeled Iba1+ cells in both males and females, suggesting that they may engage in both proinflammatory and anti-inflammatory processes. This finding contrasts the single timepoint measures of greater numbers of amoeboid microglia (typically associated with proinflammatory roles) found in males and suggests that microglia may remain multifunctional during early development [40].

**Figure 5.**
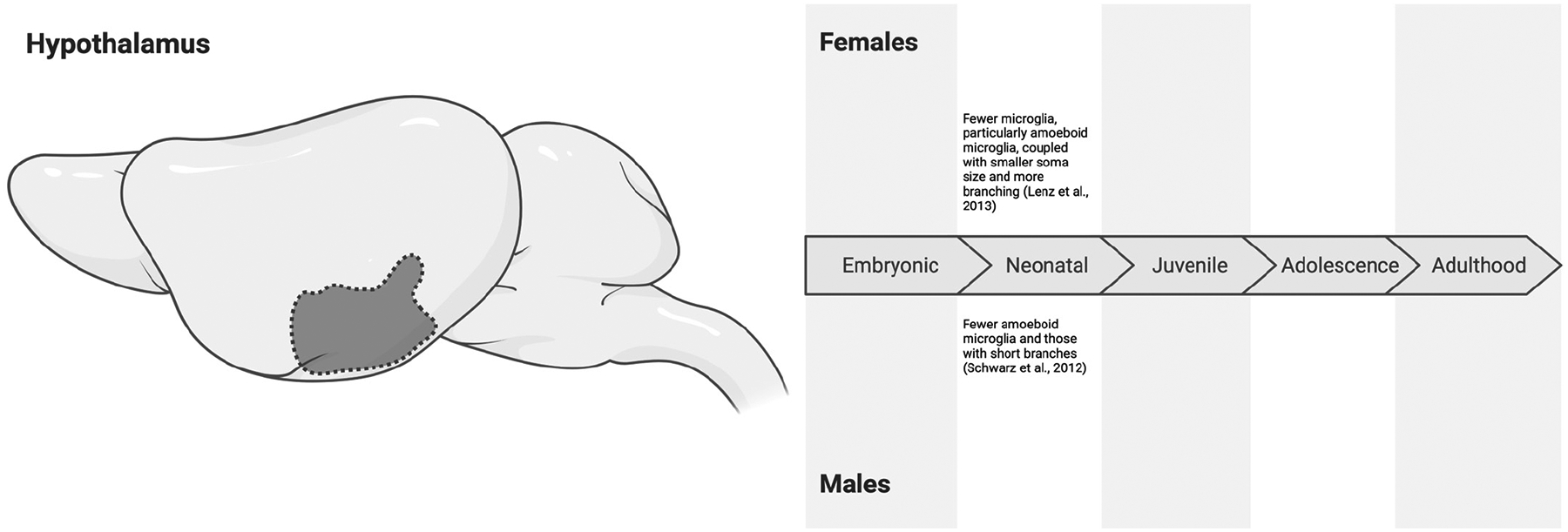
Sex differences in microglial characteristics across the embryonic (embryonic day 0 to birth), neonatal (postnatal day 0 to 10), juvenile (postnatal day 11 to 20), adolescent (postnatal day 21 to 59), and adulthood (postnatal day 60 onwards) developmental stages in the hypothalamus of mice and rats. All effects are in comparison to opposite-sex conspecifics. Transient sex differences have been observed during the neonatal period [40,78], but research on the other developmental stages is particularly lacking. Created in BioRender. Bishnoi, I. (2024) https://BioRender.com/t84z394 (accessed on 3 December 2024).

Given the highly endocrine-centric functions of the hypothalamus, there is a significant opportunity to further explore sex differences in microglial morphology, and especially function, in hypothalamic subregions. Thus far, we have seen that sex differences in morphology may not be clear at baseline until P0 in the PVN, though these differences soon dissipate. Within the POA, we are only starting to understand that microglial cells may remain multifunctional in early development (Figure 5), and potential sex differences may be mediated by estradiol. Whether basal sex differences persist into adulthood and their potential functional implications in hypothalamic subregions is yet to be uncovered.

### 2.5. The Basal Ganglia

The basal ganglia are a group of interconnected subcortical nuclei that are essential for motor function, motor learning, and reward learning, like social play behaviors [101–103]. Microglia within the basal ganglia have been implicated in neurodegenerative diseases such as Parkinson’s and Huntington’s disease [104,105], as well as Alzheimer’s disease and substance use disorders [106,107]. Emerging evidence suggests that microglia in the basal ganglia exhibit distinct characteristics that vary with subregion, sex, and age, potentially influencing their roles during development and disease.

De Biase et al. [45] examined the microglial phenotypes across the basal ganglia in male and female mice. They specifically compared the nucleus accumbens (NaCC), ventral tegmental area (VTA), substantia nigra pars reticulata (SNr), and the substantia nigra pars compacta (SNc). Compared to the other subregions, the SNr contained a lower density of microglia at P6 but experienced an explosion in microglial density less than a week later at P12. Similar increases in microglial density were seen within the SNc and NaCC, with the smallest increase seen in the VTA [45]. Compared to the SNc, NaCC, and VTA, the SNr also has the highest microglial lysosome content, suggesting greater phagocytic activity or a higher metabolic state [45]. Correspondingly, microglia in the SNr displayed significantly more hyperpolarized resting potentials and a larger membrane capacitance, indicating a more activated or reactive baseline state compared to regions like the SNc and VTA [45]. While sex differences were not statistically analyzed, these findings highlight within-region variation in microglial characteristics.

Microglia in other regions of the basal ganglia may also exhibit distinct characteristics. For example, microglia in the striatum of both male and female mice appear to preferentially engage in lower clearance activity. Ayata et al. [106] found that this phenotype may be guided by the PRC2 complex, which suppresses genes that promote clearance activity [106]. This function appears to be critical for the homeostasis of striatal microglia, as the disruption of microglial PRC2 (i.e., increasing clearance activity in the striatum) impairs the function and morphology of striatal neurons, leading them to resemble Alzheimer’s disease pathology [106]. Although Ayata et al. [106] also did not statistically analyze sex differences, they did sex- and age-match their groups. Striatal microglia of male and female adult mice have been found to differentially express 454 transcripts (288 of which were expressed at higher levels in males, and 166 were expressed at higher levels in females) [70], suggesting that microglial sex differences may exist within this region. Despite differential gene expression, Ayata et al. [106] and others [55] have found limited sex differences in microglial function and morphology in the striatum of both early adolescent and adult mice, indicating a gap that needs to be explored further.

Other studies in which sex-stratified effects were evaluated on P20, P30, P38, and P54 (though statistical comparisons between males and females were not performed), female Sprague Dawley rats had greater microglial density in the NaCC on P20, which reduced and stabilized at this reduced level in adolescence (P30-P54) [102]. In male rats, microglial density remained heightened on P20 and P30 but also reduced and stabilized into later adolescence (P38-P54). Intriguingly, CD68 staining suggested that, of these microglia, the largest amount of phagocytic microglia were found on P30 in both males and females. That said, the amount of phagocytic microglia in the NaCC of males at P38 was similar to their P30 levels. In contrast, females began to show an increase in phagocytic microglia in late adolescence on P54 [102]. Kopec et al. [102] also suggested that the sex-specific effects of microglia may mediate male-biased social play behaviors in adolescence.

During adulthood (P90), female C57BL/6J mice exhibit greater microglial density and branching complexity in the globus pallidus and substantia nigra (including both SNr and SNc) compared to males [108]. In the globus pallidus, microglia of female mice also exhibit greater soma sizes and branching complexity than males [108], leading one to speculate that microglia in the globus pallidus of female mice may play an especially active, immune-responsive role (though functional assays remain to be conducted). In other regions, like the putamen of the basal ganglia, there is currently limited evidence for sex differences in microglial characteristics in adulthood [108].

**Figure 6.**
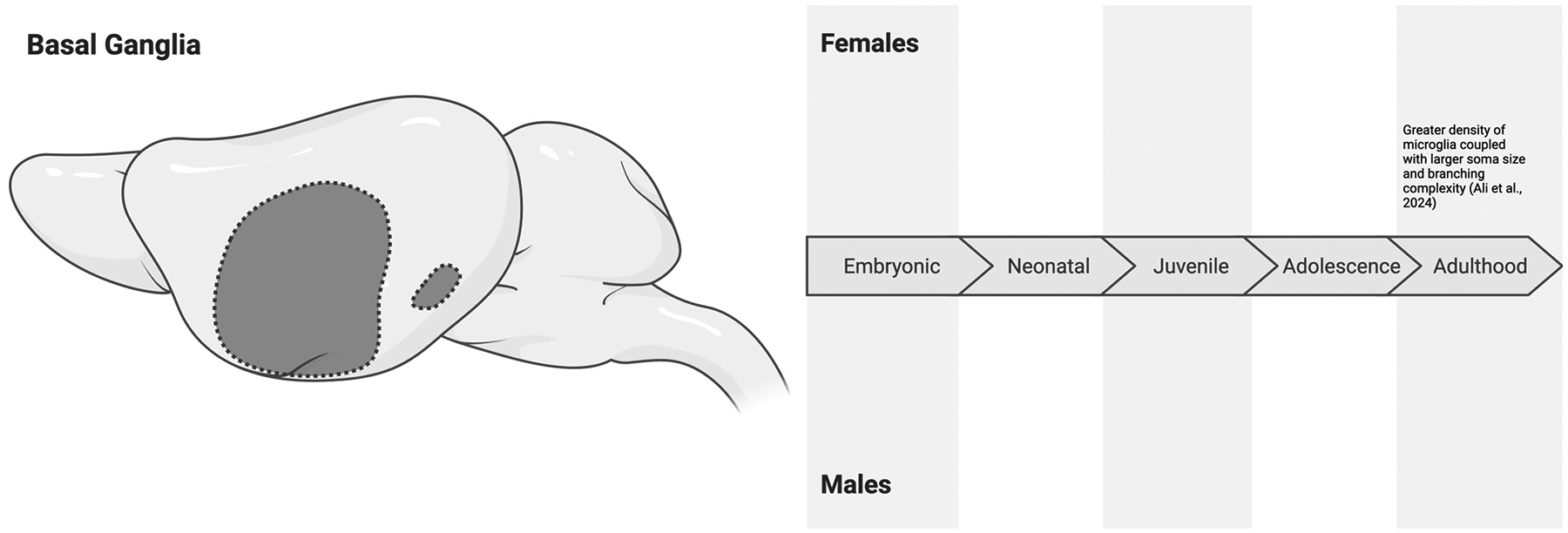
Sex differences in microglial characteristics across the embryonic (embryonic day 0 to birth), neonatal (postnatal day 0 to 10), juvenile (postnatal day 11 to 20), adolescent (postnatal day 21 to 59), and adulthood (postnatal day 60 onwards) developmental stages in the basal ganglia of mice and rats. All effects are in comparison to opposite-sex conspecifics. Many studies on region-specific effects of microglia within the basal ganglia do not statistically evaluate sex differences. When sex differences are evaluated, morphological variations are observed in adulthood [108]. Created in BioRender. Bishnoi, I. (2024) https://BioRender.com/w81x526 (accessed on 3 December 2024).

In summary, given that the basal ganglia are a group of nuclei, much of the research characterizing microglia within this area has compared between regions of the basal ganglia. This approach has provided significant insights into within-region differences which are lacking in other regions of the brain. Through these studies, we are starting to understand that sex differences may become more pronounced during adulthood, especially in specific regions like the substantia nigra and globus pallidus (Figure 6). As sex has not been included within the statistical models of some of the reviewed studies, there is a significant opportunity to reproduce these effects across the many regions of the basal ganglia. These investigations are especially important given the somewhat disparate findings when sex is included as a biological factor.

### 2.6. The Cerebellum

The cerebellum is an evolutionarily ancient structure that is traditionally associated with motor coordination and balance but also contributes to higher-order functions [109]. Cerebellar microglia are notably involved in clearing apoptotic cells and cellular debris, likely due to the high rate of cell turnover in this region during development [106]. Dysfunction in this role may particularly contribute to neurological disorders involving motor dysfunction and cerebellar neurodegeneration [110]. Sex differences in these functions are only starting to emerge.

Perez-Pouchoulen et al. [81] neatly compared sex differences in microglial morphology in Sprague Dawley rats on P17 across the molecular and granular layers of the cerebellum. They observed a significantly higher number of microglia with thin branches in the granular layer of the cerebellum in males compared to female conspecifics [81]. No significant sex differences were found in the molecular layer of the cerebellum [81]. Perez-Pouchoulen et al. [81] also analyzed sex differences in the frequency of phagocytic cups but found no differences across both the molecular and granular layers on P17.

Other studies similarly report minimal sex differences in cerebellar microglial characteristics. For instance, Guneykaya et al. [55] found no sex differences in microglial density or soma size in adolescence (P21) in C57BL/6J mice. Cealie et al. [111] corroborated these findings, observing no sex differences in total microglial density in early adulthood (P60). Interestingly, slightly later in adulthood (P77–P84), subtle sex differences in cerebellar microglial protein expression emerge. Specifically, C57BL/6 male mice express higher levels of CD40, TREM-2b, and CXCR3 and lower levels of CD86 and F4/80 compared to females [112]. These findings indicate that cerebellar microglia may be tuned to more clearing and phagocytic functions compared to maintaining homeostasis in adulthood, especially in males. Further into adulthood (P91), morphological analyses of microglia suggest an absence of these effects, with no sex difference in microglial density or soma size [55]. However, it is unclear whether this is due to age or if these morphological indicators are not fully indicative of functional states.

**Figure 7.**
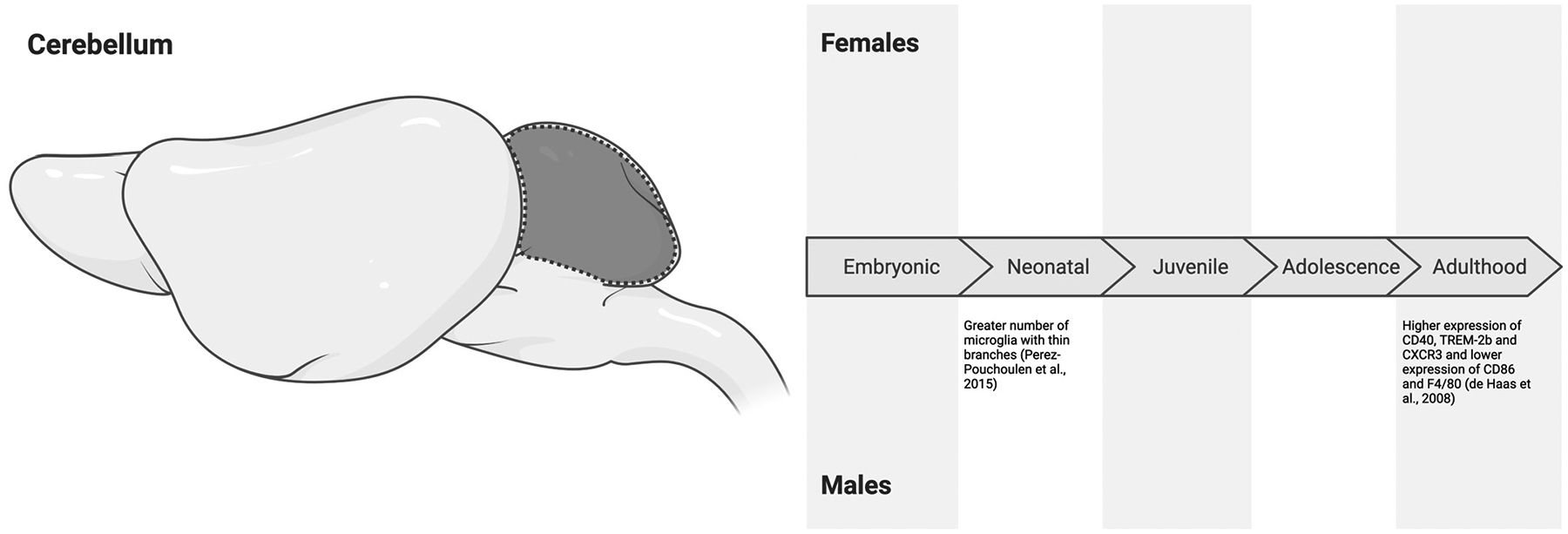
Sex differences in microglial characteristics across the embryonic (embryonic day 0 to birth), neonatal (postnatal day 0 to 10), juvenile (postnatal day 11 to 20), adolescent (postnatal day 21 to 59), and adulthood (postnatal day 60 onwards) developmental stages in the cerebellum of mice and rats. All effects are in comparison to opposite-sex conspecifics. While some studies report sex differences during the neonatal period [81], other measures suggest a lack of sex-dependent variations. In adulthood, microglia in males may play more phagocytic roles [112]. Created in BioRender. Bishnoi, I. (2024) https://BioRender.com/q92y700 (accessed on 3 December 2024).

In summary, like many of the regions discussed in this review, the cerebellum has a paucity of research on microglial characteristics during the embryonic period. Much of this issue may come down to the technical challenges associated with conducting research during this period [113–115]. When studies have explored sex differences in cerebellar microglia, limited differences have been found. While some studies, such as Perez-Pouchoulen et al. [81], report morphological sex differences according to some measures during the neonatal period, other measures suggest a lack of sex-dependent variations. To our knowledge, baseline characterizations of microglia in the adolescent cerebellum have not been performed. During adulthood, we see a shift to more phagocytic microglia in males through more functional indicators (e.g., CD40, TREM-2b, and CXCR3) but not through morphological indicators (Figure 7). Several questions remain regarding the sex differences in microglial morphology and function across development in the cerebellum. Measuring these baseline variations can give us a better starting point for understanding the shifts from baseline that may give rise to neurological disorders and neurodegeneration.

## 3. Conclusions

Microglia exhibit truly remarkable functional and morphological diversity. They are highly influenced by factors such as developmental stage, sex, hormonal fluctuations, and the unique demands of specific brain regions. The findings synthesized in this review emphasize how these factors contribute to a mosaic of microglial characteristics across the brain, highlighting the complexity of these cells within the CNS.

As evidenced throughout this review, there is significant spatiotemporal heterogeneity in microglial morphology and function. For example, the hippocampus, amygdala, and hypothalamic POA display notable sex-specific activity immediately after birth [39,40,78]. These effects may be driven by the early-life surge in androgenic and estrogenic signals [18,19,39,40,79]. A new layer of complexity is introduced during the critical period of adolescence, as hormonal surges around P40 amplify sex differences [22,23], which seem to persist into adulthood in several brain regions. In the frontal cortex and hippocampus, microglia in females tend to exhibit more ramified morphologies, whereas microglia in males within these regions display morphological and functional markers of heightened immune readiness, including increased soma size, antigen-presentation capacities, and heightened responses to ATP [55,78]. In adulthood, sex differences remain evident and region-dependent. Microglia in females within certain brain areas, such as the frontal cortex and hippocampus, continue to prioritize a homeostatic role, while microglia in males across the hippocampus and amygdala often display phagocytic characteristics. Notably, many regions exhibit age-dependent shifts, with regions presenting with sex differences in microglial activation during early development that then reverse (e.g., in the hippocampus) or disappear (e.g., in the PVN of the hypothalamus) by adulthood. In other areas, such as the cerebellum and basal ganglia, sex-dependent microglial variations are comparatively subtle or underexplored, pointing to critical gaps in our current knowledge. Addressing these gaps is critical to better recognize when these characteristics go awry, such as after insults that have been shown to alter microglia in a region- and/or sex-dependent manner, including maternal stress, limited bedding and nesting, intestinal dysbiosis, exposure to opioids, air pollution, infections, and much more [100,116–120].

A deeper understanding of region-specific microglial sex differences and factors that can alter microglial function has broad implications, particularly in understanding sex-biased susceptibility to neurodevelopmental and neurodegenerative disorders. There is a growing appreciation that microglial sex differences may reflect adaptations aligned with the functional roles of their local environments or brain regions. For instance, compared to microglia of the striatum of the basal ganglia, microglia of the cerebellum are highly involved in phagocytosis and clearance, which may make the cerebellum more resilient to Alzheimer’s disease pathology where an accumulation of plaques has been found to be mitigated by more active microglia [106]. This may especially serve as an advantage in older adult males, who may exhibit more active phagocytic microglia than females [112]. Indeed, there is a higher incidence of Alzheimer’s disease in older adult females than males [121,122]. This female-biased vulnerability may not be solely driven by gonadal hormones, as the incidence rate of Alzheimer’s disease increases during menopause when estrogen and progesterone are in decline [123], suggesting that these hormones may play a protective role against the progression of Alzheimer’s disease [124]. Possibly due to estrogen’s anti-inflammatory effects [65], microglia observed in females appear to be oriented towards neuroprotection. This orientation may confer other forms of resilience, such as to early-life stress or immune challenges [125], which are known to play major roles in male-biased disorders like autism spectrum disorder [126–128]. There is, however, a significant amount of research needed to understand the factors driving these sex-specific patterns of resilience and vulnerability. Going forward, accounting for the local environment of microglia in these effects will be critical [45,102].

This review highlights several key findings but also underscores many areas that remain underexplored. Firstly, the waves of gonadal hormones may not impact all brain regions in the same way. Thus, there is a significant opportunity to gain deeper insights into the story of gonadal hormones in the sex differences being observed across brain regions. Female microglia often exhibit a neuroprotective phenotype (e.g., a lower expression of inflammatory genes like Trem-1 and Cxcl2) that persists even when transplanted into male brains [129]. While traditional gonadal hormones may have a role to play in these effects, this finding may also suggest that some microglial sex differences may be independent of circulating gonadal hormones, instead relying on intrinsic cues. Thus, beyond androgen and estrogen signaling, future research should also explore the roles of other factors like the progesterone hormone, whose receptors are also found on microglia [27,29], downstream targets such as prostaglandins [40], the potential for genetic and epigenetic influences [130,131], in addition to tracking and accounting for the estrus cycle in future rodent studies.

In the present review, we primarily focused on research within rats and mice. While researchers are beginning to see similarities and differences between species, to our knowledge, there is no literature that has compared baseline microglial sex differences between species. There is additionally a need to prioritize longitudinal studies that track microglial characteristics across the lifespan, from the embryonic period through aging, across both sexes. Such studies can provide new perspectives on how microglia transition through different morphological and functional states. Longitudinal studies can also provide significant insights into sex differences in age-related cognitive decline or resilience to neurodegenerative diseases. Finally, while the current review has taken a segmented approach to outlining microglia at baseline, sex differences in microglia go beyond the discussed regions. Circuits, lesser-studied regions (such as the subventricular zone and circumventricular organs), and even specific components of the discussed regions, such as subregions of the amygdala, could also shed light on region-specific roles of microglia that influence broader CNS functions.

As researchers tackle these complex questions, the methods used to address them are equally important. It is essential to recognize that microglial morphology alone cannot fully capture their function. This is because microglia are not static. Rather, in their local environments, microglia appear to behave like dynamical systems, continuously changing their state and responsiveness from moment to moment [132]. Thus, the limitations inherent in morphological studies point to the need for comprehensive, multimodal approaches, such as combining recent advances in high-resolution and time-lapse imaging, transcriptomics, proteomics, and multiplex morphological assessments to capture the spectrum of microglial diversity [133–135]. Different outcomes may be found between these measures, such as morphological observations being inconsistent with functional findings (as we see across several of the discussed regions) or even artificial changes in outcomes [136]. However, given the dynamic nature of microglia [42], these studies can weave together stories that reshape our understanding of these beautifully complex cells.

Ultimately, advancing our understanding of sex differences in microglia will depend on developing and employing consistent methodologies and high-throughput techniques capable of capturing their diverse roles within the CNS. This knowledge is indispensable for further unraveling sex- and region-specific differences in microglial characteristics across ages. By gaining a deeper appreciation for these baseline differences, we can better recognize when they go awry. Consequently, we may be seeing the beginnings of research uncovering heterogeneous microglial characteristics, which may lead to promising avenues for the development of sex-specific therapeutic strategies for neurological and psychiatric disorders.
